# Husbandry Practices, Health, and Welfare Status of Organic Broilers in France

**DOI:** 10.3390/ani9030097

**Published:** 2019-03-19

**Authors:** Rozenn Souillard, Jean-Michel Répérant, Catherine Experton, Adeline Huneau-Salaun, Jenna Coton, Loïc Balaine, Sophie Le Bouquin

**Affiliations:** 1 ANSES, Epidemiology, Health and Welfare Unit, BP 53, 22440 Ploufragan, France; adeline.huneau@anses.fr (A.H.-S.); jenna.coton@gmail.com (J.C.); loic.balaine@anses.fr (L.B.); sophie.lebouquin-leneveu@anses.fr (S.L.B.); 2 ANSES, Virology, Immunology and Parasitology in Poultry and Rabbits Unit, BP 53, 22440 Ploufragan, France; jean-michel.reperant@anses.fr; 3 ITAB, 149 rue de Bercy, 75595 Paris, France; catherine.experton@itab.asso.fr

**Keywords:** organic, broiler, sustainability, husbandry, health, welfare

## Abstract

**Simple Summary:**

Organic poultry production has grown rapidly in Europe for the past several years in the context of sustainable development within farming. The authors carried out a field study in France between 2014 and 2015 concerning 85 organic broiler flocks that showed a wide diversity of farming management systems from independent farmers set up for direct sales of poultry to farmers under contract with a company for product sales. Health and welfare characteristics did not significantly differ between these two farming systems, except slightly dirtier feathers and more footpad dermatitis on the independent farms, related to the poultry housing conditions in mobile houses. A mortality rate of 2.8% was found, with digestive problems mainly being observed. Better knowledge of husbandry practices, health, and the welfare status of organic poultry is of primary importance to improve the management of organic production and to help in characterizing farming sustainability.

**Abstract:**

Organic poultry production has increased sharply with growing consumer demand in the context of sustainable development. A study was conducted in 85 organic broiler flocks between 2014 and 2015 to describe the husbandry practices and the health and welfare status of organic broilers in France, and to study farming diversity by comparing independent farms (Ind farms, *n* = 15) with direct sales to farms working with companies (Comp farms, *n* = 70). Each flock was visited at 3 and 11 weeks of age to collect data on farming conditions, health disorders, and mortality. Welfare notation of 30 broilers per flock and parasitic examination of 5 broilers per flock was also performed. Findings showed significantly different farming management between Ind farms and Comp farms, with smaller flocks on the Ind farms (476 broilers/house vs. 3062 broilers/house, *p* < 0.01) more frequently in mobile houses. The mean mortality rate was 2.8%, mainly involving digestive disorders. Helminths were detected in 58.8% of the flocks. On average, 21.9% and 5.8% of broilers in a flock had footpad dermatitis and dirty feathers, respectively. The health and welfare characteristics of organic broilers on Ind farms vs. Comp farms were not significantly different, except dirtier feathers and more footpad dermatitis on Ind farms (19.1% vs. 2.9%, *p* = 0.03 and 39.6% vs. 18.1%, *p* = 0.02, respectively), associated with poultry housing conditions in mobile houses (*p* < 0.01). This study provides greater insight into farming sustainability aspects related to the husbandry practices, and the health and welfare of organic broilers in France.

## 1. Introduction

Consumer interest for organic products has increased substantially in recent years. Between 2005 and 2015, a 14% yearly increase of the organic poultry head numbers occurred in Europe [1]. Organic poultry production in France has also progressed with 794 organic broiler farms in 2016, a 5% increase compared to 2015. France is the top-ranking producer of organic broilers in Europe [2]. In many countries, studies have shown the growing interest of consumers for local products, in particular for organic production [3]. The direct sale model is also developing in France, particularly in organic poultry farming [4]. Unlike in other animal production sectors, such as cattle farming, the development of direct sales seems to be easier in poultry because of the short rearing period of chickens and the possibility of having a slaughter room on the farm with whole chicken sales [4]. There is wide diversity among organic poultry producers, with independent farmers selling their product themselves only to the local market, and farmers under contract with a company for the sale of their production.

Organic farming must fulfil consumers’ expectations in terms of animal health and welfare, the environment, and food safety. Organic broiler production is based on rearing rules (Commission Regulation (EC) No. 889/2008) characterized by high demands concerning animal housing and husbandry: A high level of animal welfare with an outdoor access area, specific rearing conditions (fewer than 4800 broilers/house, minimum slaughter age of 81 days, and maximum density of 10 broilers/m^2^ in stationary houses and 16 broilers/m^2^ in mobile houses), and health management based on preventive measures with limited use of medical drugs [5].

In the context of growing criticism of intensive farming systems, the general public and consumers are showing an increased interest in the development of sustainable farming, taking into account environmental, economic, and social sustainability [6]. Given the growing worldwide demand for animal products between now and the year of 2030, particularly in the poultry sector [7], the challenges are to develop farming while reducing environmental impact, maintaining economic viability, and remaining socially responsible. Sustainability development is a multi-dimensional notion and the ways sustainability farming is implemented generate debate [8]. There are a wide range of animal production systems with advantages and disadvantages. Organic farming is based on a more environmentally friendly production and a higher animal welfare status and the conventional system involves more intensive and productive rearing conditions. The assessment of the sustainability of farming productions is complex given the various factors involved. A recent review comparing conventional and organic systems showed some differences in sustainability aspects between the two systems (concerning in particular the income per animal, impact on biodiversity, and resistance of bacteria to antibiotics). This study also highlighted a significant lack of data for assessing the sustainability of many organic animal productions [9]. Most studies on organic farming have focused on dairy cattle [9,10]. A better understanding of current organic farming production systems would help to develop more sustainable production models for organic poultry [9].

Despite the rapid increase in organic production and the challenges related to the sustainability of farming systems, very little knowledge is available on organic broiler farms. Therefore, we carried out an epidemiological study on 85 organic broiler farms in France to provide better insight into and knowledge of husbandry practices, and the health and welfare status of organic broilers, and to study the diversity of organic production systems.

## 2. Materials and Methods

### 2.1. Study Design and Farming Data Collection

This study was carried out between January 2014 and April 2015 and involved 85 organic broiler flocks in France (about 10% of the French organic broiler farms), with 70 farmers under contract with a company (Comp farmers) and 15 independent farmers (Ind farmers). The population was stratified according to commercial outlets in France: 80% of farmers were under contract with a company for production sales (chicks and food supply, poultry slaughter by the company) and 20% were independent farmers selling their production themselves to local markets for direct sale only [2]. Given the absence of a national register of organic broiler farms in France, the farmers under contract were selected from seven production companies and the independent farmers via organic producer associations. The geographical distribution of the 85 studied farms was based on the national distribution of farms in the main regions of the country [2], with 40 farms (47%) in the North-West, 14 farms (16.4%) in the South-West, and 31 farms (36.4%) in the Centre and South-East. Farm selection was also based on the farmers’ agreement to participate in the study.

The epidemiological unit in this study was a single flock of organic broilers per farm, with more than 250 chickens. Each flock was visited twice, at 3 weeks of age (before outdoor access) and at 11 weeks of age (the end of the rearing period). The visits of the farms were conducted separately by 2 investigators. During an interview with the farmers, a questionnaire was filled in to collect data regarding the farm, the poultry house, the flock management, and the potential health disorders (Table 1). The health disorders reported by the farmers were diagnosed as usual by their veterinarian’s technical support. The farmers were also asked to record the number of dead birds daily. This mortality included culled and dead birds due to health problems, but does not take into account other losses, such as deaths by predators. In addition, a bacteriological analysis of the drinking water was carried out in each broiler house (*Escherichia coli*, sulphite-reducing anaerobes, and *Enterococcus*). The investigators took water samples at the end of the water line. These samples were analysed in accredited laboratories according to the AFNOR (National standardization organization) standards. After the slaughter of the broiler flocks, the farmers were contacted to collect specific slaughtering data.

### 2.2. Parasite Examination

A parasite examination was carried out to investigate the presence of coccidia, coccidial lesions, and helminths. This parasite examination was performed during each visit on 5 broilers per flock: At 3 weeks of age, a critical period for coccidiosis, and at 11 weeks of age at the end of the rearing period, a relevant period to find helminths. The 5 broilers were randomly chosen in the house and sacrificed on the farm (electronarcosis and bleeding) and the digestive tract was individually sampled and brought to our laboratory of parasitology. This protocol was approved by the animal-welfare body of ANSES (French Agency for Food, Environmental and Occupational Health & Safety). Coccidial lesions were investigated in the small intestine and ceca according to the method described by Johnson and Reid [11] for the three main species of coccidia in chickens: *Eimeria acervulina, Eimeria maxima*, and *Eimeria tenella* (Table 2). The coccidia were identified under a microscope, after scraping the intestinal and cecal mucosal membrane.

For the detection of helminths, the two main ascarids of poultry, *Ascaridia galli* and *Heterakis gallinarum*, were screened. Direct examination of the intestinal content was performed to look for *Ascaridia galli*, and filtering of cecal content though a 1 mm diameter sieve followed by observation on a black background was carried out to screen for *H. gallinarum.*

### 2.3. Welfare Indicators

During each visit, welfare indicators were assessed in 30 randomly chosen broilers per flock according to Welfare Quality^®^ scoring [12]: Feather with score 0: Clean, score 1: Minimal degradation, score 2: Dirty, and score 3: Very dirty; footpad dermatitis and hock lesions with score 0: No lesions, score 1: Minimal lesions, and score 2 to 4: Evidence of lesions according to their severity (2: Mild, 3: Severe, and 4: Very severe). The photographs of the Welfare Quality^®^ protocol were also used as a reference for the scoring of the feather and the hock and footpad dermatitis lesions. Litter quality was scored during each visit in each quarter of the house (3 notations per quarter in watering, feeding, and resting areas), according to score 1: Dry litter (does not stick to boots) and score 2: Wet litter (sticks to boots).

### 2.4. Data Processing

Data collected on farming conditions, and the health and welfare characteristics were described as the mean (with SD: Standard deviation) or frequency for the 85 flocks and for the two strata (*n* = 15 Ind farms vs. *n* = 70 Comp farms). The results were also compared between these two strata using χ^2^ test for qualitative data and non-parametric Wilcoxon test for quantitative data, considering a significant difference with *p* < 0.05. This data processing was performed with statistical software R (R foundation, Vienna, Austria).

## 3. Results

### 3.1. Organic Broiler Farms Characteristics

Independent farmers started organic broiler production on the farm more recently, on average 11 years ago (SD = 4.8) vs. 14.5 years (SD = 5.8) for the farmers in companies (*p* = 0.03). The organic broiler flocks were more commonly raised under multi-age conditions (without all-in-all-out procedure) on the independent farms (14/15 vs. 23/70 on farms in companies, *p* < 0.01). The number of organic broiler flocks/year was higher on independent farms, on average 10.6 flocks (SD = 5.1) vs. 3.7 flocks (SD = 2) on farms in companies (*p* < 0.01).

### 3.2. Housing Conditions of the 85 Organic Broiler Flocks

The housing conditions of the 85 organic broiler flocks are shown in Table 3. The housing conditions on independent farms differed markedly from farms in companies. On the independent farms, broiler flocks were raised more frequently in mobile houses moved onto fields (10/15 vs. 18/70, *p* < 0.01), with significantly smaller areas (40.3 m^2^ vs. 294.4 m^2^) and flock sizes (476 broilers vs. 3 062 broilers). Ventilation and food distribution were more commonly manual on the independent farms compared to the farms in companies (14/15 vs. 43/70, *p* = 0.05 for ventilation, and 15/15 vs. 9/70, *p* < 0.01 for feed). The type of range was more frequently a meadow on the independent farms (10/15 vs. 25/70, *p* = 0.03). The other types of ranges had planting with hedges or trees.

The mobile houses were characterized by specific housing conditions with manual regulation of ventilation (27/28 mobile houses), the ground on a field for all of them, and a mean density of broilers significantly higher than in stationary houses (14.9 broilers/m^2^ (SD = 1.9) vs. 9.9 broilers/m^2^ (SD = 0.5)). The litter was also wetter in mobile houses. A wetter litter (at least one score = 2) was observed in visit 1 and in visit 2, respectively, in 5/28 (18%) mobile houses vs. 8/57 (14%) stationary houses and in 11/28 (39%) mobile houses vs. 17/57 (29%) stationary houses, with no significant difference.

### 3.3. Husbandry Practices in the 85 Organic Broiler Flocks

Husbandry practices are shown in Table 3. The husbandry practices on independent farms differed from those on farms in companies. Feed for the start of the rearing period was commonly made at the farm by the independent farmers (9/15 vs. 5/70, *p* < 0.01). No significant difference was observed for drinking water management, except for the hygiene treatment of drinking water, which was more frequently used by the farmers in companies (5/15 vs. 46/70, *p* = 0.02). Regarding prophylaxis practices, the flocks on independent farms were less vaccinated against viral infection (Gumboro disease, infectious bronchitis, or Newcastle disease) (8/15 vs. 67/69, *p* < 0.01), and the independent farmers also used less preventive treatments for their flocks (on average 0.9 vs. 2.1, *p* = 0.03). The preventive treatments were mainly phytotherapy products and nutritional supplements (vitamins and minerals). Most of the flocks were vaccinated against coccidiosis (87%), without a significant difference between the independent farms and the farms in companies.

The organic broiler flocks on independent farms were commonly slaughtered in an approved slaughter room on the farm (7/15 vs. 0/70, *p* < 0.01). Given the slaughtering of small numbers of animals to supply the local market, few slaughtering data were recorded on the independent farms and it was not possible to collect them. Data regarding the slaughter age and weight of organic broilers were obtained for about 60 flocks on the farms in companies (despite contacts with the farmers): The chickens were slaughtered at an average of 86 days (SD = 5.6) (*n* = 60 flocks), and the mean slaughter weight was 2.4 kg (SD = 0.19) (*n* = 62 flocks).

### 3.4. Animal Health Characteristics

Health disorders were reported by the farmers in 32 flocks, with no significant difference between the independent farms (3/15 flocks) and the farms in companies (29/70 flocks) (Table 4). Health problems were primarily digestive disorders in 24 flocks: 18 cases of unspecified enteritis on 3 independent farms and 15 farms in companies, and 4 cases of necrotic enteritis and 2 cases of coccidiosis on farms in companies. Coccidiosis outbreaks occurred in two flocks although animals were vaccinated against the disease. Five farmers in companies used an antibiotic to treat necrotic enteritis in two flocks, arthritis with *Staphylococcus aureus* in two flocks, and a respiratory disorder associated with mortality in one flock. To treat a coccidiosis outbreak, one farmer also used an anticoccidial drug. The other farmers used only alternative products, mainly based on plants. The mean mortality rate at 77 days of age was 2.8% (SD = 2.7%) in the 85 flocks, and 3.1% vs. 2.7%, respectively, for the flocks on independent farms (*n* = 9 flocks) and the flocks on farms in companies (*n* = 68 flocks), with no significant difference.

### 3.5. Parasite Examination

Coccidia were observed in most of the 85 flocks at visit 1 and visit 2, with no significant difference between the independent farms and the farms in companies (Table 4). Few coccidial lesions (with minor score, Reid scoring ≤ 2) were observed at visit 1 and at visit 2, independently from the independent vs. farms in companies’ strata. At visit 1, the lesions were associated with *E. acervulina* (13 flocks out of 24 with coccidial lesions), *E. maxima* (5 flocks), *E. tenella* (3 flocks), and *E. maxima* and *E. tenella* (3 flocks). The two cases of coccidial lesions observed at visit 2 were associated with *E. tenella*.

Larvae of helminths were identified at visit 1 in two flocks before access to the outdoor range (*Heterakis* on one independent farm and *Ascaridia* on one farm in a company). At visit 2, helminths were identified in 58.8% (50/85) of the flocks. The presence of helminths tended to be more frequent in flocks on independent farms (12/15 vs. 38/70, *p* = 0.07). On the independent farms, the helminths observed at visit 2 were *Heterakis* in six flocks and *Heterakis* and *Ascaridia* in six flocks. For the flocks on farms in companies, *Heterakis* was observed in 21 flocks, *Ascaridia* in 10 flocks, and *Heterakis* and *Ascaridia* in 7 flocks. Helminths in poultry were more frequently identified in the case of a wetter litter in the house (at least one score = 2) (*p* < 0.01): Parasites were detected in 78% (22/28) of the houses with wet litter vs. 49% (28/57).

### 3.6. Welfare Status

In visit 1, only minimal degradation (score = 1) of the poultry feathers was observed for on average 1.9% (SD = 7.3) of broilers per flock. Footpad dermatitis at visit 1 (score ≥ 2) were observed on average in 5.1% (SD = 13.2) of broilers per flock. These welfare indicators in visit 1 did not significantly differ between independent farms and farms in companies (Table 4). For the hock lesions, very few were observed with only minimal lesions (score = 1) at visit 1 in three flocks (with less than 2 broilers/30).

In visit 2, the mean percentage of broilers in a flock with dirty feathers (score ≥ 2) at visit 2 was 5.8% (SD = 16.6), and was higher for the flocks on independent farms compared to the farms in companies: 19.1% vs. 2.9%, respectively *p* = 0.03 (Table 4). Footpad dermatitis at visit 2 (score ≥ 2) was observed on average in 21.9% of broilers/flock (SD = 25.9), and more frequently in the flocks on independent farms than on farms in companies, respectively, 39.6% vs. 18.1%, *p* = 0.02. For the hock lesions, very few were observed with only minimal lesions (score = 1) at visit 2 in five flocks (with less than 5 broilers/30).

The observation of dirty feathers and footpad dermatitis was associated with the housing conditions of poultry in mobile vs. stationary houses (*p* < 0.01): On average, 8.4% of broilers per flock with dirty feathers in mobile houses vs. 4.5% in stationary houses, and 37% of broilers per flock with footpad dermatitis in mobile houses vs. 14.4% in stationary houses. More dirty feathers and footpad dermatitis were observed in the case of a wetter litter (at least one score = 2) (*p* < 0.01): On average, 12% of broilers per flock had dirty feathers on a wet litter vs. 2.7%, and 32.4% of broilers per flock had footpad dermatitis on a wet litter vs. 16.7%.

## 4. Discussion

### 4.1. Study Design

Organic production includes a sustainable approach to farming, considering environmental, economic, and societal challenges. Further knowledge on many specific aspects of sustainability is needed to help the future development of sustainable farming [9]. This study was designed to improve insights on the husbandry practices, and the health and welfare status of organic broilers in France, and to study the diversity of organic poultry production. Although the farmers were not randomly selected, the final population can be considered to be representative of organic broiler farms in the country. The sampled farms represent a significant proportion with more than 10% of the French organic broiler farms and this number of flocks was also included in a study considering logistical and laboratory constraints. The 85 farms in our study follow the distribution of national organic chicken production in France [2]. In addition, 17.6% of the farmers in our study were independent farmers, which is comparable to the 20% independent organic chicken farmers in France [2]. We were able to verify the representativeness of our sample by comparing some farming data with those available in France on 49 organic broiler flocks on farms in companies [13]. These flocks are comparable to our study population (number of flocks/year and slaughtered weight of the broilers were on average 3.3 vs. 3.7 and 2.5 kg vs. 2.4 kg, respectively, in our study). The national proportion between independent farms and farms in companies was maintained in our study population to cover a representative sample of organic broiler production in France, and, at the same time, to compare these two organic broiler farming systems. The survey questionnaire and the protocol of observations were tested and standardized before implementation, and data were collected by only two investigators. Any assessment bias during observations can therefore be considered limited. The assessment of the feather, the footpad dermatitis, and hock skin lesions was based on the Welfare Quality^®^ protocol. However, a wide range of other welfare indicators are included in Welfare Quality^®^, like mortality or litter quality. In our study, the mortality was reported daily by the farmer and the litter quality was scored according to our protocol. The same referential for all the indicators would have given a more standardized picture of the welfare status.

### 4.2. Housing Conditions and Husbandry Practices

This study showed a wide diversity in farming management practices between the independent farmers and the farmers in companies. The management of the flocks on the independent farms was suitable for direct sales, with a smaller size of flocks (on average 476 boilers vs. 3 064 on farms in companies) raised in multi-age conditions, and commonly slaughtered in a slaughter room on the farm, allowing for permanent availability of products. The housing conditions of the flocks on the independent farms, usually in mobile houses, seem to be related to management with less automation, particularly with the manual regulation of ventilation and the distribution of feed. The independent farmers also used significantly fewer preventive products (phytotherapy products or nutritional supplements). According to a survey studying the perception of health management by 18 organic broiler farmers [14], a lack of technical support was mentioned by the independent farmers, contrary to the farmers in companies who rely mainly on the technicians and veterinarians of their company.

### 4.3. Animal Health Characteristics

This study shows that the health status of organic broilers on independent farms is not significantly different from that of organic broilers on farms in companies, with digestive disorders mainly being observed, with the detection of helminths in 58.8% of the flocks, and a mortality rate of 2.8% at 77 days of age. The few available studies on health problems in organic broilers [15,16] also show that digestive disorders appear to be the main problems in this production [15,17]. Importantly, necrotic enteritis and coccidiosis was reported in organic broilers in Denmark [15]. The risk of coccidiosis is expected to be higher in organic broilers. The coccidiostats in the feed are not allowed in organic production and the disease frequently occurs in the absence of preventive treatment [15]. Vaccination against coccidiosis is therefore recommended on organic farms, otherwise this disorder would be the main health problem [17]. Indeed, the majority of the flocks in our study were vaccinated against coccidiosis, and only two coccidiosis outbreaks were reported. These two coccidiosis outbreaks occurred in two flocks that were vaccinated against the disease. Outbreaks of coccidiosis may occur in vaccinated poultry because of various possible factors (improper storing and vaccine administration, bad vaccine recycling, or concomitant pathogens interfering with the onset of protective immunity).

The anticoccidial vaccinations based on the administration of live attenuated coccidia to chicks (indistinguishable from wild coccidia strains) would explain why coccidia were detected in most of the flocks in our study, with minor coccidial lesions. Regarding helminths, worms (Ascaridia and/or Heterakis) were detected in more than half of the organic broiler flocks (58.8%) in our study. Intestinal helminths affect free-range birds in particular [18,19,20]. This is due specifically to the high resistance of helminth eggs in the environment and to the possible intervention of intermediate hosts [21]. In Sweden, a higher prevalence of Ascarid eggs in faecal samples was indeed reported in free-range organic laying hens (77.1%; *n* = 2 free-range and 32 organic flocks) compared to hens in cages (4.3%; *n* = 46 furnished cages) [18]. Regarding broilers, Ascarid infections are not generally a problem in conventional production because of indoor housing and the short rearing period [21], and very few data are available regarding worm infections in organic production. In Belgium, worms were screened for on nine organic broiler farms (faecal examination for worm eggs at slaughter in 1 g of fecal sample from 30 broilers) [16], but none were identified. This result could be explained by the low number of flocks investigated and the screening method. The clinical influence of helminths in poultry is generally low. However, depending on the worm burden, they can affect the growth and health of poultry. *Ascaridia galli* can lead to reduced weight gain, and heavy infestations can cause diarrhea and anorexia [22]. *A. galli* eggs may also act as mechanical vectors of salmonella [20]. *H. gallinarum* causes very few clinical signs, but is an important vector of *Histomonas meleagridis*, causing histomoniasis. Consequently, the control of helminths in organic poultry, especially based on grazing management strategies to break the life cycle of parasites, is of primary importance to maintain healthy rearing conditions [20]. The control of helminths is also based on biosecurity practices and litter management. The optimal conditions for the development of eggs are wet and cold conditions [22]. In our study, helminths were more frequently identified in organic broilers in the case of a wetter litter in the house. Concerning mortality in the organic broiler flocks, the mean mortality rate of 2.8% for the 85 flocks in our study at 77 days of age is related to the health problems of broilers up to 11 weeks of age, and does not take into account other losses, such as deaths by predators. This could explain the higher mortality rates in organic broiler flocks reported in other studies, with 5.5% in Denmark [15] and 4.1% in France [13].

### 4.4. Welfare Status

The welfare characteristics of organic broilers were significantly different between the independent farms and the farms in companies, with statistically more broilers/flocks with dirty feathers on independent farms (on average 19.1% vs. 2.9%), and with footpad dermatitis (on average 39.6% vs. 18.1%). The physical health of the animals and their behaviour are the main aspects of animal welfare [23], covering all the farming conditions and the phases of the poultry chain from the breeder to slaughter. In organic production, animal welfare also includes the concept of naturalness, which is the expression of species’ behaviour in relation to animal physiology and the natural environment [24]. The observation of skin integrity (hock burn or footpad dermatitis) is one of the ways to evaluate the welfare status of birds [23]. Lesions that were the most relevant to broilers and that were easy to identify on the farms were scored in our study according to Welfare Quality^®^. Footpad dermatitis is most commonly reported in conventional systems associated with the more intensive rearing conditions of poultry (particularly higher stocking density and faster growth rates) [25,26], with widely variable incidences of 13% [27], 48.9% [28], or more than 90% [29]. Tuyttens et al. showed that organic birds had better hock condition, leg health, and overall welfare than those in conventional production systems [30]. However, it was also reported that access to an outdoor area could influence the development of footpad dermatitis, which is probably explained by the fact that birds raised outside would be more likely to sustain foot injuries on the range that may cause skin damage [31,32]. It is difficult to compare these findings on the welfare status of broilers because of the very varying scoring systems, samples used, and the various rearing conditions of poultry. Many farming factors can indeed influence footpad dermatitis. Footpad dermatitis are caused by wet litter, resulting in skin lesions, and several factors can exacerbate lesions, particularly environmental factors (litter material, ambient conditions, or stocking density), nutritional factors (nutritional deficiencies), and poultry characteristics (strain, body weight, or gut health) [33]. In our study, more dirty feathers and footpad dermatitis were significantly observed in the case of a wetter litter. A higher frequency of dirty feathers and footpad dermatitis was reported on the independent farms. This could be explained by the housing conditions of poultry in mobile houses commonly used on independent farms. Dirty feathers and footpad dermatitis are significantly associated with mobile houses, and certain specific housing conditions described in our study could promote them: The ground on a field probably causing wetter litter, manual regulation of ventilation probably making ambient management more difficult, and a higher broiler stocking density. A wetter litter was also observed in mobile houses compared to the stationary houses without a significant difference. Given the large number of factors that may influence the development of footpad dermatitis, further research is needed to study more specifically the rearing conditions associated with the welfare status of organic broilers.

## 5. Conclusions

The aims of this study were to describe the husbandry practices and the health and welfare status of organic broilers in France, and to study farming diversity by comparing independent farms with direct sales to farms working with companies. This study provides better insights into and knowledge of sustainability aspects related to organic broiler farming, a growing sector of poultry production in Europe. The sampled farms of our study cover a significant proportion of organic broiler production in France with more than 10% of the French organic broiler farms. Nevertheless, our study presents a limitation related to the standardization of the welfare indicators that could have been based on the same referential.

Sustainability is a complex concept, including economic, environmental, and social issues. This study highlighted the farming characteristics related to the direct sale of organic broilers in France and a wide diversity in faming management practices between independent farms and farms under contract with a company for sale of their production. The local market is a farming model in development in many countries. This model could be part of environmental sustainability development, providing the possibility to reduce transport and its environmental impact. This study also provides a large overview of the welfare and health status of organic broilers in France, which enhanced the knowledge of social sustainability farming aspects. A better characterization of organic broiler production helps the future development of sustainable organic farming.

Further research is needed to identify the impact of rearing conditions on the health and welfare status of organic broilers. An epidemiological model could be proposed to explain the health and welfare status of organic broilers with regards to structural farming factors and husbandry management factors. Such a study could provide elements to enhance the management and sustainability of organic broiler production.

## Figures and Tables

**Table 1:** Summary of the items included in the survey questionnaire.

Farm characteristics	Farmer activity
	Organic broiler production on the farm
	Management of the poultry flocks
Housing conditions	Structure of the house
	Food distribution
	Ventilation
Range	Type of the range
	Management of the range
Husbandry practices	Characteristics of the flock Litter management
	Feeding
	Watering
Health context	Health disorders reported
	Curative treatment
	Mortality
Prophylaxis	Vaccination
	Preventive treatment
Slaughtering	Slaughterhouse
	Slaughtering data

**Table 2:** Coccidial lesions according to the method described by Johnson and Reid [11].

Species of Coccidia	Score 0	Score 1	Score 2	Score 3	Score 4
*E. acervulina*	No gross lesion	A few white spots on the mucosal surface of the duodenum	Lesions much closer together but not coalescent, which may extend to 20 cm in the jejunum	Coalescent lesions and watery content.	White aspect of the mucosal surface. No feed, watery content. White creamy exudate.
*E. maxima*	No gross lesion	Small red petechiae on the serosal side of the mid-intestine	Numerous petechiae. Possible orange mucus	Ballooning and thickening of the intestine. Much orange mucus and watery content.	Ballooning of all the intestine. Orange mucus, blood clots and digested blood.
*E. tenella*	No gross lesion	Very few petechiae on the cecal wall. Pasty cecal content.	More petechiae. Cecal wall somewhat thickened. Blood mixed with cecal content.	Cecal wall greatly thickened. Only blood or blood cores.	Cecal wall greatly distended with blood or fibrinous cores.

**Table 3:** Housing conditions and husbandry practices of the organic broiler flocks (*n* = 85, France, 2015).

Category	Characteristics	Independent Farms *n* = 15	Farms in Companies *n* = 70	*p*-Value
House	Broiler flock size *	476 (216)	3062 (1222)	<0.01
	Type of house			<0.01
	Mobile	10 (66.7%)	18 (25.7%)	
	Stationary	5 (33.3%)	52 (74.3%)	
	House area m^2^ *	40.3 (17.4)	294.4 (142.4)	<0.01
Range	Type of range			0.03
	Meadow	10 (66.7%)	25 (35.7%)	
	Range with planting	5 (33.3%)	45 (64.3%)	
	Age access to range in days *	39.9 (6.8)	42.3 (4.7)	0.27
	Range area m^2^ *	2404 (1560.9) *^b^*	15447 (8371.9) *^c^*	<0.01
Ventilation regulation	Manual	14 (93.3%)	43 (61.4%)	0.05
	Partial automatic	1 (6.7%)	18 (25.7%)	
	Total automatic	0	9 (12.9%)	
Feeding	Feed distribution			<0.01
	Manual	15 (100%)	9 (12.9%)	
	Automatic	0	61 (87.1%)	
	On farm feed production	9 (60%)	5 (7.1%)	<0.01
Drinking water	Private water supply	5 (33.3%)	16 (22.9%)	0.39
	One water analysis/year	3 (20%)	23 (32.9%)	0.33
	Compliance microbiological criteria	1 (6.7%)	14 (20%)	0.22
	Water hygiene treatment	5 (33.3%)	46 (65.7%)	0.02
Prophylaxis	Viral vaccination	8 (53.3%)	67 (97.1%) *^a^*	<0.01
	Coccidial vaccination	11(73.3%)	63 (90%)	0.13
	No. of preventive treatments *	0.9 (0.9)	2.1 (1.9)	0.03
Slaughter of broilers flocks	Slaughterhouse	8 (53.3%)	70 (100%)	<0.01
	Slaughter room on the farm	7 (46.7%)	0	

* mean (standard deviation); *^a^*: 1 missing data point; *^b^*: 3 missing data points; *^c^*: 4 missing data points.

**Table 4:** Health and welfare characteristics of the organic broiler flocks (*n* = 85, France, 2015).

Category	Characteristics	Independent Farms *n* = 15	Farms in Companies *n* = 70	*p*-Value
Health disorders reported	Health disorders	3 (20%)	29 (41.4%) *^a^*	0.12
	Digestive disorder	3 (20%)	21 (30%)	
	Locomotor disorder	0	3 (4.3%)	
	Early rearing disorder	0	4 (5.7%)	
	Respiratory disorder	0	2 (2.8%)	
Mortality	Mortality rate (77 days of age) *^b^* *	3.1% (2.3)	2.7% (2.7)	0.66
Parasite (*n* = 5 broilers/flock)	Coccidia			
	Visit 1	13 (86.7%)	63 (90%)	0.70
	Visit 2	13 (86.7%)	49 (70%)	0.19
	Coccidial lesions			
	Visit 1	5 (33.3%)	19 (27.1%)	0.63
	Visit 2	1 (6.7%)	1 (1.4%)	0.22
	Helminths			
	Visit 1	1 (6.7%)	1 (1.4%)	0.22
	Visit 2	12 (80%)	38 (54.2%)	0.07
Welfare indicators (*n* = 30 broilers/flock)	Feathers			
	Visit 1 (% broilers/flock—score = 1) *	3.6% (8.3)	1.5% (7.1)	0.18
	Visit 2 (% broilers/flock—score ≥ 2) *	19.1% (33.1)	2.9% (7.9) *^c^*	0.03
	Footpad dermatitis			
	Visit 1 (% broilers/flock—score ≥ 2) *	7.6% (13.5)	4.6% (13.2)	0.24
	Visit 2 (% broilers/flock—score ≥ 2) *	39.6% (33.5)	18.1% (22.5) *^c^*	0.02

* mean (standard deviation); *^a^* two diseases reported in one flock in company (unspecified enteritis and omphalitis); *^b^* *n* = 9 independent flocks and *n* = 68 flocks from farms in companies; *^c^* one missing data point.
